# Concurrent Takayasu Arteritis and Vascular Ehlers–Danlos Syndrome: A Case Report

**DOI:** 10.3389/fcvm.2022.805505

**Published:** 2022-01-26

**Authors:** Kyota Hashimoto, Ryota Sakai, Akiko Shibata, Yusuke Okada, Syoichi Yoshinaga, Takahiko Kurasawa, Tsuneo Kondo, Koichi Amano

**Affiliations:** ^1^Clinical Training Center, Saitama Medical University Hospital, Saitama, Japan; ^2^Department of Rheumatology and Clinical Immunology, Saitama Medical Center, Saitama Medical University, Saitama, Japan

**Keywords:** Takayasu arteritis, vascular Ehlers-Danlos syndrome, neutrophil, tocilizumab, interventional radiology

## Abstract

Takayasu arteritis (TAK) is a rare primary systemic inflammatory vasculopathy. It is classified as a large-vessel vasculitis and is known to cause inflammatory aneurysms and vascular stenosis. Vascular Ehlers–Danlos syndrome (vEDS) is an autosomal dominant condition known to cause multiple aneurysms and arterial dissection at a young age owing to a mutation in the gene for type III collagen, *COL3A1*. Here, we present a case of TAK associated with vEDS with the development of multi-organ infarction of the brain, kidney, and spleen owing to multiple arterial aneurysms and stenosis of the internal carotid artery. The patient was successfully treated using anti-inflammatory agents, glucocorticoids, and tocilizumab with the addition of interventional radiology. In our case, a high inflammatory response led to vasculitis being the main cause of the disease with concurrent vEDS. When patients develop multiple aneurysms, stenosis, and dissections leading to multiple organ infarctions, a systemic differential diagnosis to consider concurrent vasculitis syndrome and non-inflammatory vasculopathy, including hereditary disorders, is important even with time constraints.

## Introduction

Takayasu arteritis (TAK) is a rare primary systemic inflammatory vasculopathy of unknown etiology. It is classified as a large-vessel vasculitis (LVV), predominantly affecting the aorta and/or its major branches; however, arteries of any size may be affected (1). Inflammatory aneurysms and stenosis depending on the vascular wall thickening are common features of this disease. There are no specific laboratory diagnostic markers; however, a high inflammatory response, such as a high C-reactive protein (CRP) level and specific radiographic findings, are helpful for the diagnosis. Recently, tocilizumab (TCZ), a humanized anti-interleukin (IL)-6 receptor (IL-6R) monoclonal antibody, is a promising agent for TAK when administered in combination with glucocorticoids (GCs) for the remission-induction therapy (2). Ehlers–Danlos syndrome (EDS) is a hereditary disorder that affects connective tissues, such as the skin, joints, and blood vessel walls. The International Consortium on Ehlers–Danlos syndrome and related disorders proposed an updated EDS classification in 2017, which presently recognizes 13 subtypes (3). One of these subtypes, vascular Ehlers–Danlos syndrome (vEDS), is known to cause multiple aneurysms and arterial dissection at a young age. This is inherited in an autosomal dominant manner and is due to a mutation in the gene for type III collagen, *COL3A1*. Midsize arteries are the most commonly involved, and arterial rupture is the most common cause of sudden death. Genetic sequencing is the gold standard for diagnosing vEDS; however, obtaining genetic results is time-consuming, which adds to the ethical consideration. In urgent situations, clinicians are required to diagnose and treat diseases based on limited information. Herein, we report a case of TAK associated with vEDS with the development of multi-organ infarction of the brain, kidney, and spleen due to multiple aneurysms and stenosis of the internal carotid artery. The patient was successfully treated by anti-inflammatory agents, GCs, TCZ, and interventional radiology (IVR).

## Case Report

A 28-year-old Japanese man was admitted to our hospital with hypovolemic shock secondary to abdominal hemorrhage and hemiplegia due to a left cerebral infarct. Thirteen days prior to his admission, he developed a right lower abdominal and back pain that worsened in the prone position. Seven days before admission, he had a fever of 38°C and had visited another hospital where his serum CRP level was 13.8 mg/dL (normal range, 0–0.15 mg/dL). Since contrast-enhanced computed tomography (CT) showed a splenic and right renal infarction with multiple aneurysms of the hepatic, right renal, left common iliac, and right internal iliac arteries, and no intra-abdominal bleeding was observed, he was started on anticoagulant therapy, heparin, on admission at that hospital for a week. On the morning of his admission, he experienced undifferentiated dizziness and aggravated abdominal pain. His systolic blood pressure was noted to be in the 60s, and intra-abdominal bleeding from multiple aneurysms was observed on abdominal CT. He was rushed to our emergency room for hypovolemic shock due to bleeding from that hospital.

**Figure 1:**
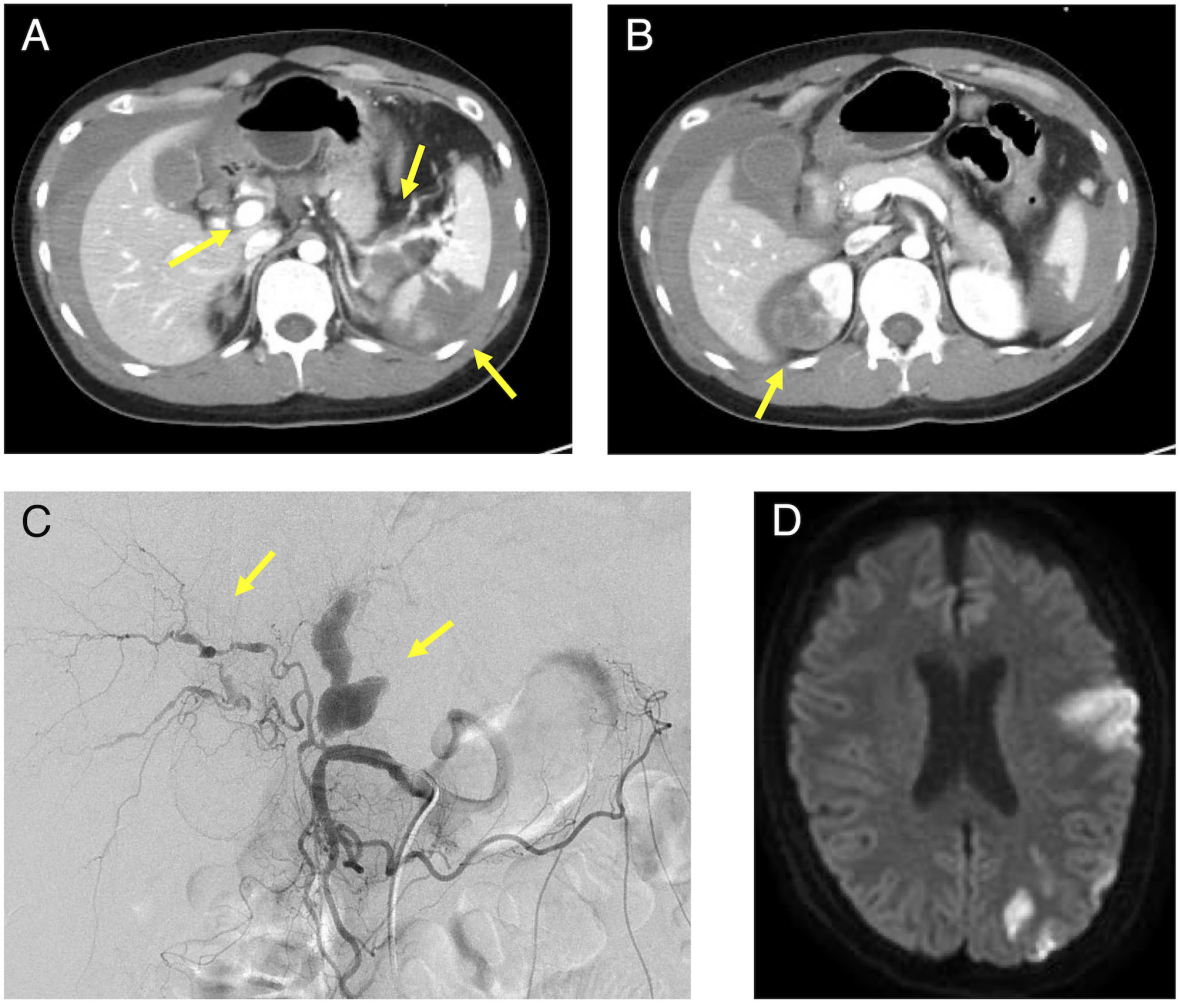
Contrast-enhanced CT, angiography, and head MRI of the patient. **(A)**: Intra-abdominal hemorrhage, splenic infarction, and hepatic and splenic artery aneurysm were observed in the area indicated by the arrow and **(B)**: Right renal infarction was observed in the area indicated by the arrow on the contrast-enhanced abdominal CT. **(C)**: A proper hepatic artery aneurysm was observed in the area indicated by the arrow on angiography. **(D)**: A cerebral infarction was observed in the left middle cerebral artery territory on the plain MRI of the brain. MRI, magnetic resonance imaging; CT, computed tomography.

**Figure 2:**
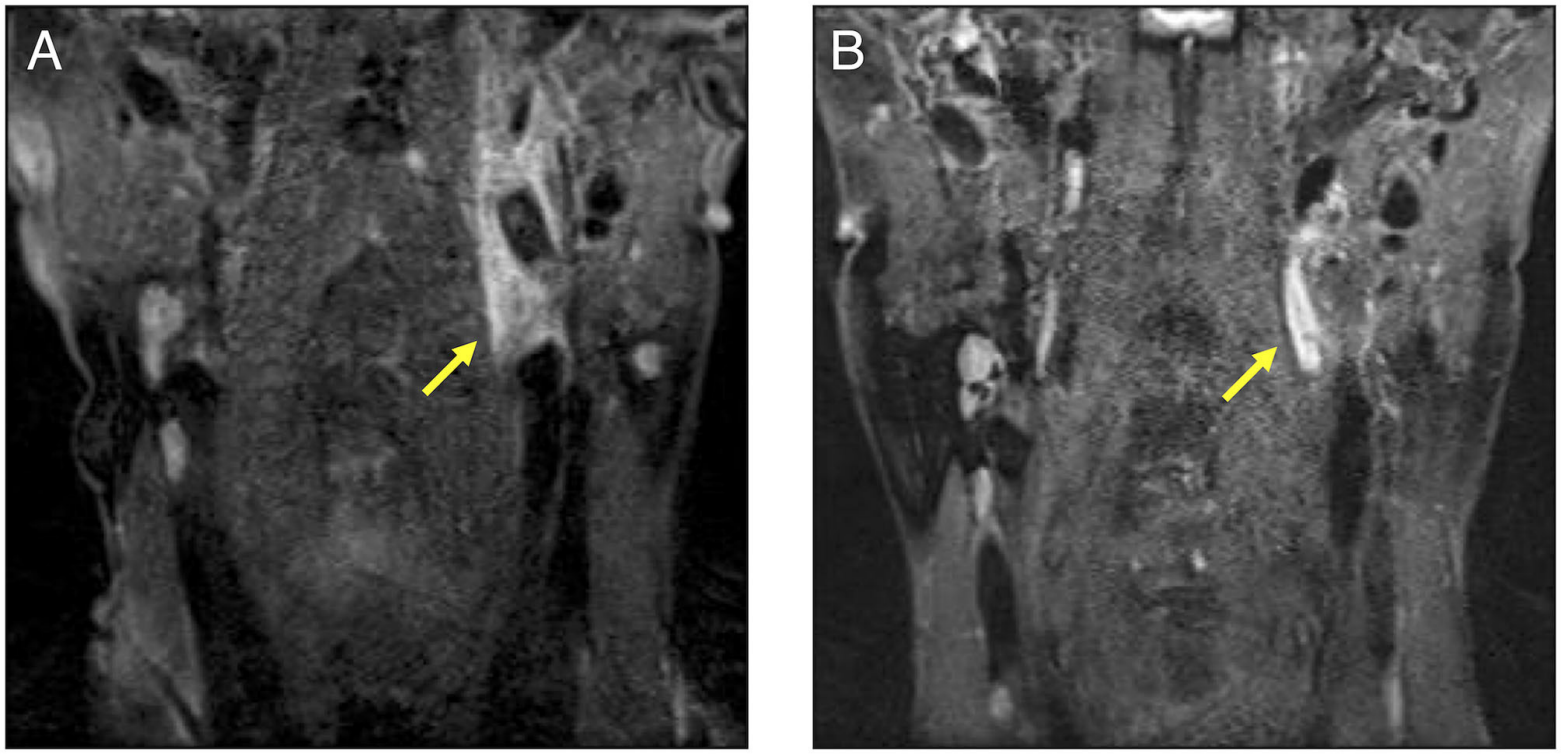
A cervical contrast-enhanced MRI comparison before and after the treatment in our patient. **(A)**: This MRI was performed before treatment. Aneurysm, stenosis, and dissections were observed in the left internal carotid artery indicated by the arrow. A contrast effect was also observed around the lesion. **(B)**: The participant underwent MRI as an outpatient after treatment. The contrast effect around the left internal carotid artery had decreased. MRI, magnetic resonance imaging.

His medical history included bronchial asthma in childhood, ventricular septal defect, osteoma ossificans, and herpes zoster. Additionally, his family history included a grandmother with spinocerebellar degeneration, a grandfather with hypertension, a father with rheumatoid arthritis and dyslipidemia, and a mother with diabetes mellitus. Further, there was no family history of EDS, other genetic diseases, or sudden cardiovascular death in relatives. The patient showed no difference in blood pressure in the upper extremities. The neurological findings included a complete paralysis of the right upper and lower limbs and dysarthria. No other findings were observed in the remaining physical examinations. Blood tests revealed a markedly elevated inflammatory response with a white blood cell count of 38,000/μL (neutrophil: 99%, lymphocyte: 1%) and CRP of 5.02 mg/dL. All autoantibodies and blood culture test results were negative. A transthoracic cardiac ultrasound revealed no vegetation. Contrast-enhanced CT and angiography showed multiple aneurysms of the hepatic, splenic, right renal, left common iliac, and right internal iliac arteries, as well as infarction of the spleen and right kidney with intra-abdominal bleeding (Figures 1A–C). The hepatic aneurysm was enlarged compared to those taken at another hospital 1 week prior (from 13.6 × 7.8 to 16.5 × 13.0 mm). No vascular occlusion was observed on the contrast-enhanced CT scan. Contrast-enhanced magnetic resonance imaging (MRI) of the head and neck showed infarction of the left middle cerebral artery region (Figure 1D) and left internal carotid artery aneurysm, stenosis, and dissection (Figure 2A). Contrast enhancement was observed around the stenotic area; however, a positron emission tomography (PET) scan of the entire body 1 week after starting the treatment revealed no abnormalities. In other words, rupture of the aneurysm leading to intra-abdominal bleeding and stroke-induced progression of vascular stenosis were observed in a short period. IVR was performed shortly after admission. Embolization was performed to treat the hepatic and splenic artery aneurysms, and the bleeding stopped. Although vEDS was a differential diagnosis, the patient had a severe inflammatory response, probably due to vasculitis.

**Figure 3:**
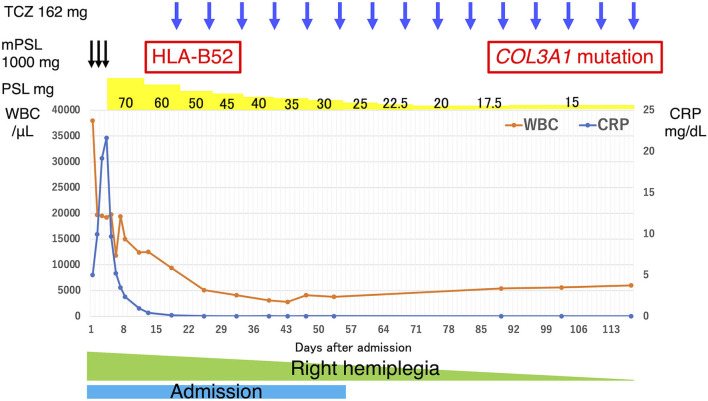
Clinical course of our patient. The vertical axis of the graph represents the WBC count and CRP levels, and the horizontal axis represents the number of days since admission. The drug content, dosage, dosing interval, and results of the generic tests are noted above the graph. The duration of admission and hemiplegia are shown in the graph. CRP, C-reactive protein; TCZ, tocilizumab; mPSL, methylprednisolone; PSL, prednisolone; WBC, white blood cell.

We administered intravenous methylprednisolone (mPSL) [1,000 mg/day] for 3 days, followed by prednisolone (PSL) at a dose of 70 mg/day (1.2 mg/kg), with the dose reduced every 1–2 weeks (Figure 3). Human leukocyte antigen testing (HLA) showed B52, which is common in patients with TAK. Following the administration of GCs, 162 mg of tocilizumab was subcutaneously administered weekly. The inflammatory response and paralytic symptoms improved gradually. No relapse after discharge from our hospital was observed on administering low dose GCs and weekly TCZ. When a neck MRI was performed again in the outpatient clinic after 6 months of remission-induction therapy, there was a decrease in the contrast effect and an improvement in the internal carotid artery stenosis (Figure 2B). Although there were no physical findings and family history consistent with vEDS, a follow-up CT scan revealed a dissection of the celiac trunk and the right femoral artery, which was associated with IVR. Finally, the patient was tested for *COL3A1* gene mutation, and a positive result was obtained.

## Discussion

To the best of our knowledge, this is the first case report demonstrating TAK associated with vEDS with a combination of inflammatory and non-inflammatory underlying causes and the subsequent development of multi-organ infarcts of the brain, kidney, and spleen due to multiple arterial aneurysms and stenosis of the internal carotid artery. Despite the urgency and the critical situation, we could arrive at a conclusive diagnosis by adequately performing physical examinations, imaging radiography, and blood tests. In addition, there have been no reports of catheter-based aneurysm embolization in the acute stage of TAK and vEDS, as observed in this case. Causes of non-atherosclerotic peripheral arterial stenosis and aneurysm, such as polyarteritis nodosa and Behçet's disease (BD), were considered differential diagnoses for systemic vasculitis. In contrast, segmental arterial mediolysis (SAM), infectious aneurysms, and hereditary elastic fibrous disorders, such as EDS, Loeys–Dietz syndrome, and Marfan syndrome, were considered differential diagnoses for non-inflammatory vasculopathy (4). The key to diagnosis in such cases includes performing an adequate physical examination, the presence of significantly elevated inflammatory response markers in blood tests, and specific radiographic findings. Consequently, we diagnosed TAK based on the high inflammatory response, positive HLA-B52, perivascular inflammation and stenosis, and multiple arterial aneurysms. Previous research has uncovered the genetic components involved in the pathogenesis of TAK, and HLA-B52:01 is the only definite genetic factor globally (5–8). One reason for observing no abnormalities on the PET scan, in this case, was because it was taken 1 week after treatment initiation; the inflammation could have disappeared. Second, due to poor resolution on PET scan, most of the affected vessels were medium-sized in this case and could, thus, not be detected by PET scan. Although we cannot deny the possibility that the patient developed an inflammatory response due to bleeding or infarction, it does not explain the inflammatory findings around the left internal carotid artery observed on contrast-enhanced MRI. In addition, there was no rupture or enlargement of the abdominal aneurysm after the immunosuppressive therapy for ~1 year.

In addition, the concurrent diagnosis of vEDS was established based on the genetic results of the *COL3A1* gene mutation. An analysis of a previous study in Japanese patients with vEDS revealed a low frequency of young patients presenting with serious clinical findings, such as arterial rupture/dissection/aneurysm and perforation or rupture of the gastrointestinal tract (5). However, our patient developed a severe intra-abdominal hemorrhage and organ infarction, possibly due to vasculitis complications. Although the presence of vEDS does not change the treatment, the complication of vEDS could lead to rapid progression of rupture or stenosis, as in this case, and late therapeutic intervention could have resulted in a fatal outcome. Moreover, aneurysms were observed in the medium-sized vessels; therefore, polyarteritis nodosa was also a high priority in the differential diagnosis in this case. Although mutations in the adenosine deaminase 2 gene are known to cause polyarteritis nodosa in young people (9), genetic analysis performed in our patient yielded negative results. A notable feature of this case was the marked increase in neutrophils at the onset of vasculitis. Neutrophils and neutrophil extracellular traps (NETs) contribute to the pathogenesis of systemic vasculitis, including small and medium-sized vasculitis and LVV (10). Therefore, the elevation of neutrophils may be consistent with vasculitis due to TAK in this case. In addition, BD, which is characterized by recurrent oral and/or genital aphthous ulcers accompanied by cutaneous, ocular, articular, gastrointestinal, central nervous system inflammatory, and/or vascular lesions (1), was also one of the important differential diagnoses in this case. However, all these characteristics are not necessarily present, as apparent in our case. Vascular BD is characterized by blood vessels with a neutrophil-dominating infiltration around the vasa vasorum, compared with inflammatory aneurysms and TAK (11). Therefore, neutrophils may have induced rupture or dissections of aneurysms in this case. In contrast, SAM, a non-inflammatory vascular disorder, was excluded since a systematic review concluded that the median age of onset for this disease is 57 years (range, 0–91 years). Furthermore, catheterization alone often improves the disease status because of its non-inflammatory nature (12), which is inconsistent with our case. In addition, results that might be consistent with a diagnosis of an infected inflammatory aneurysm were not observed.

EDS is a group of 13 hereditary connective tissue diseases characterized by decreased joint mobility, hyperextension of the skin, and tissue fragility. Additionally, vEDS is a type of EDS, causing aneurysms and arterial dissection in juveniles. Shalhub et al. emphasized the importance of confirming *COL3A1* mutations for the diagnosis of vEDS (13). Although there was no family history of vEDS in this patient, it has been reported that only 48.8% of the 86 patients who tested positive for *COL3A1* had a positive family history, suggesting that vEDS cannot be excluded based on family history alone (14). Specifically designed small interfering RNAs (siRNAs) can effectively silence the pathogenic variant allele of *COL3A1*. To enhance normal allelic expression, intracellularly expressed lysyl oxidase can regulate the transcription of the *COL3A1* promoter (15). Hence, genetic mutations cannot necessarily regulate gene expression, which can change the phenotype. In addition, a study that analyzed genetic mutations and clinical manifestations of vEDS suggested that specific *COL3A1* mutations may not be associated with the complications of this disease phenotype (16, 17). However, it has similarly been suggested that the probability of severe complications, such as ruptured aneurysms, increases with age, emphasizing the importance of a follow-up (17).

A recent study reported a case of EDS-hypermobility type combined with TAK; however, there is no information on TAK associated with vEDS (18). In another study, Caudrelier et al. reported a case of a young woman with vEDS and polyarteritis nodosa (19). The patient had multiple arterial aneurysms, mainly in medium-sized vessels, and was treated with steroids, cyclophosphamide, and azathioprine. This report suggests that vEDS and vasculitis may coexist and can be treated by anti-inflammatory agents, such as GCs, and immunosuppressive agents. In our case, genetic results for vEDS were delayed and were received after treatment with remission-induction therapy. The delay in diagnosis and treatment could have led to catastrophic results; however, the patient was successfully treated with anti-inflammatory agents, GCs, TCZ, and IVR. Therefore, cases of multiple arterial aneurysms and dissections with an inflammatory response should be considered for treatment with anti-inflammatory drugs to reduce the progression of inflammation.

## Conclusion

It is pivotal to perform a systemic differential diagnosis to consider concurrent vasculitis syndrome and non-inflammatory vasculopathy, including hereditary disorders, even within time constraints in patients with multiple aneurysms, stenosis, and dissections, as this could lead to multiple organ infarctions of undetermined cause.

## Data Availability Statement

The original contributions presented in the study are included in the article/supplementary material, further inquiries can be directed to the corresponding author/s.

## Ethics Statement

Ethical review and approval was not required for the study on human participants in accordance with the local legislation and institutional requirements. Written informed consent was obtained from the relevant individual for the publication of any potentially identifiable images or data included in this article.

## Author Contributions

KH: wrote the draft of the manuscript and described the figures. RS: conceived, designed, wrote the manuscript, and treated the patient as a primary attending physician. AS, YO, SY, TKu, TKo, and KA: contributed to the patient's clinical care, writing the manuscript, and providing fruitful advice. All authors have read and approved the final version of the manuscript.

## Conflict of Interest

KA has received research grants from Asahikasei Pharma Corp. and Chugai Pharmaceutical Co., Ltd., and has received speaking fees from AbbVie GK, Astellas Pharma Inc., Chugai Pharmaceutical Co., Ltd., Eisai Co., Ltd., Eli Lilly Japan K.K., GlaxoSmithKline, Janssen Pharma, and Pfizer Japan Inc. The remaining authors declare that the research was conducted in the absence of any commercial or financial relationships that could be construed as a potential conflict of interest.

## Publisher's Note

All claims expressed in this article are solely those of the authors and do not necessarily represent those of their affiliated organizations, or those of the publisher, the editors and the reviewers. Any product that may be evaluated in this article, or claim that may be made by its manufacturer, is not guaranteed or endorsed by the publisher.
